# Comment on: ‘Blood does not buy goodwill: allowing culling increases poaching of a large carnivore’

**DOI:** 10.1098/rspb.2016.1459

**Published:** 2017-03-22

**Authors:** Kim M. Pepin, Shannon L. Kay, Amy J. Davis

**Affiliations:** United States Department of Agriculture, National Wildlife Research Center, Animal and Plant Health Inspection Service, Wildlife Services, 4101 Laporte Avenue, Fort Collins, CO 80521, USA

Chapron & Treves [1] present a framework for examining effects of wolf culling policies on wolf population growth rate. They develop a population growth model that estimates an effect of the amount of time per year legal culling is allowed (‘policy effect’) on wolf population growth rates, separate from an effect of culling. They infer that there is substantial evidence for a negative relationship between the proportion of the year that the culling policy is in effect and the population growth rate because 83% of the posterior distribution for the policy effect parameter was negative. They conclude that when it is legal to cull wolves, their population growth rate is slower than it would be when it is not legal to kill wolves, even after accounting for effects of culling on population growth rates. By considering additional analyses showing that the levels of legal culling are not causing negative density-dependence, they argue that wolf culling policies devalue wolves in the public's eye such that poaching activity increases. We have several major issues with the conclusions drawn from this work.

First, the magnitude of the policy effect is biologically weak, but the biological significance (impact to the wolf population) was not presented or discussed in [1]. To show the biological significance, we plotted predictions from the model [1] with and without the policy effect included (figure 1). If the policy effect is biologically meaningful, there should be substantially fewer wolves in the model that includes the policy effect relative to one that does not. However, when the policy effect is included, predicted abundance from the two models did not appear to be biologically meaningful to wolf population growth rate, with an average of −7.8 wolves different per year that the policy was in place ([−28.2, 5.5] range for 95% credible intervals), which is on average 1.5% of the population ([−5.8%, 1.1%] range for 95% credible intervals).

**Figure 1.**
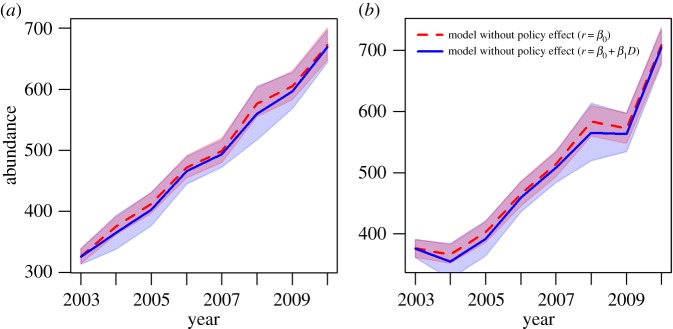
Predictions from population model [1]. We used the median parameter values (and 95% credible intervals) presented in the electronic supplementary material and the model specified in the text of [1] to plot the wolf abundance trajectories with and without the policy effect. (*a,b*) Each line represents the mean of 1000 stochastic predictions from the model (where abundance ∼ lognormal (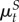, *σ*_proc_), and *σ*_proc_ was the median value estimated in [1]). We used *N*_obst−1_ as the initial condition because the authors did not provide numerical estimates of *N_t_*_−1._ Thick red dashed lines: median values of *N_t_* for the model without the policy effect (*r* = *β*_0_) for (*a*) Michigan and (*b*) Wisconsin; shaded red region: 95% credible interval of red dashed line; thick blue line: median values of *N_t_* for the model with the policy effect (*r* = *β*_0_ + *β*_1_*D*); shaded blue region: 95% credible interval of blue line. Note that the 95% credible interval for the model with the policy effect overlaps that of the one without the policy effect almost completely, indicating that the models are not substantially different.

Second, Chapron & Treves argued that because 83% of the probability distribution for the policy parameter is negative, and only 17% is positive, there is substantial evidence that the policy effect is driving growth rate to be lower than it would be without the policy in place. However, we disagree that this is a substantial negative effect. Seventeen per cent is a high rate of type 1 error, suggesting that there is considerable evidence that the relationship could be spurious and in fact driven by a correlated extrinsic factor or pure noise. We recognize that strict adherence to type 1 errors of 5% is impractical in ecology and agree that results should be considered in terms of strength of evidence. Considering the hypothesis-testing approach used in [1], there is still a 1 in 5 chance the policy effect is positive.

To explain our disagreement with the statistical interpretation of [1], we adopted a multimodel inference approach, which is well accepted in ecology [2], by comparing models with and without the policy effect. First, we implemented the model [1] (our methods shown in electronic supplementary material, S1.1 and R code) to verify we were capturing the same results as [1] (electronic supplementary material, S2). In doing so, we discovered the model specified in [1] had a typo—the correct specification (which we implemented) is: 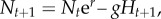 based on the organization of the data provided in the R file [1]. We simulated two scenarios (methods shown in electronic supplementary material, S1.2) using the same proportional overlap with 0 in the posterior distribution as [1] (i.e. 83% negative, 17% positive): a strong biological effect (figure 2*a*) and a weak biological effect (figure 2*b*). We evaluated the statistical support for policy effects using 80% credible intervals (to be liberal—only 80% confident about the range of uncertainty) on models with and without the policy effect. For the strong biological effect, when the distribution overlaps 0 by 17%, even though the biological effect appears strong, the 80% credible intervals overlap the model with no policy effect completely, and thus these two models are not substantially different (figure 2*a*). A similar situation can be seen when the biological effects are the same magnitude as in [1] (weak; figure 2*b*). By contrast, when the posterior distribution is more precise (overlaps 0 by 1%), results that have strong statistical support can be obtained under weak or strong biological effects (figure 2*c,d*). However, under weak biological effects, even when the posterior distribution is 99% negative, the credible intervals of the two models still overlap considerably and the models do not appear substantially different. For these reasons, when biological effects are weak it is especially important to apply several different methods for evaluating effects, such as model comparison as we have done here. We calculated a likelihood-based statistic, the deviance information criterion (DIC), for the models with and without the policy effect, and found they are statistically indistinguishable (table 1), indicating that the model without the policy effect is the most parsimonious. Lastly, we found that the direction of the policy effect differed by state (table 1), although the effects for both states were not biologically (or statistically) significant.

**Figure 2.**
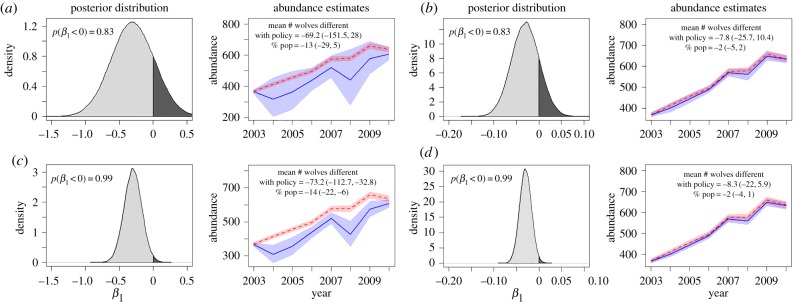
Strength of biological and statistical effects. Fits for models with (blue) and without (red) the policy effect (*β*_1_) using posterior distributions of the policy parameter which overlap 0 by (*a,b*) 17% (as in [1]) versus (*c,d*) 1%. For each level of overlap (17% versus 1%) we contrasted hypothetical results for a (*a,c*) strong and (*b,d*) weak biological effect. To be liberal with our allowance of type I error, shaded regions are 80% credible intervals (20% type I error for a two-tailed hypothesis, 10% for a one-tailed hypothesis). When the biological significance is weak, the models are not substantially different even when the posterior distribution barely overlaps 0.

**Table 1.** Biological and statistical results for models of wolf population growth with and without the policy effect. Here we are predicting mean number of wolves different between the two models using parameters that were estimated by each model. In figure 1, we only had parameters from the model with a policy effect, thus predictions for the model without the policy effect use parameters from the model with the policy effect but with *β*_1_ set to 0. This explains the discrepancy in mean wolves different between table 1 and paragraph 2 in the main text.

			mean no. wolves diff. with policy (95% CI)		
description	model on growth (*r*)	DIC	Michigan	Wisconsin	posterior of policy effect (95% CI)
policy effect	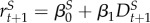	475.74	−2.01 (−4.46, −1.11)	1.08 (−0.19, 2.59)	*β*_1_ = −0.03 (−0.19, 0.12)
no policy effect	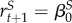	474.96	n.a.	n.a.	n.a.

In addition to comparing models with and without policy effect, we explored alternative model specifications (electronic supplementary material, S3 and S4, and table S2), some of which included density-dependent population growth. In the logistic models, the median parameter for the policy effect was 0.003 ([−0.28, 0.27] range for 95% credible intervals) and the DIC values between the models with and without the policy effect were not substantially different (electronic supplementary material, S4 and table S2), further indicating lack of support for a substantial policy effect.

Our last major concern is that there is no evidence for the source of the potentially negative effect, yet it is strongly inferred to be increased poaching (i.e. title of the work). The authors do consider one alternative explanation: negative density dependence. The hypothesis is that culling could slow growth by a mechanism other than the numbers removed because there could be a lag in reproductive response that depends on population density. Because the authors find no evidence for this process, the conclusion is that the policy effect must be due to increased poaching. However, there were no data on poaching rates for testing the poaching hypothesis. We fully agree that it is useful to discuss ideas for underlying causes of results that are best supported by knowledge of the system, because discussing results helps guide future research to rigorously test hypotheses. However, it is misleading to draw a strong conclusion based on a hypothesis that is untested.

Putting our major issue about over-interpretation of results aside, we have additional concerns with the reasoning against negative density-dependent processes in [1]. For one, the idea was not thoroughly tested due to lack of data and the result does not agree with previous work showing density-dependent reproductive processes [3–7], which should be discussed. Additionally, the factors tested (pack size, pack reproduction probability and area occupied by packs) were not given equal consideration as the policy effect (i.e. they were covariates of abundance, not growth rate—the main process in the model). Also, from a biological standpoint, it is unclear how these factors would in fact drive negative density dependence. The estimate associated with the area covered by packs was 0 ± 0, yet we found that area covered by packs was more than 95% correlated with abundance, suggesting there should have been some non-zero association with abundance.

Whatever the explanation for a potential policy effect, it should be tested with data in order to draw a solid conclusion about the mechanistic effects of the policy, as indicated in the title of a study. From our perspective, the authors [1] test the null hypothesis—‘A policy that allows wolf culling by the government causes no effects on wolf population growth rate beyond the number of wolves removed from policy actions’—and do not reject it.

We agree that management decisions should be based on rigorous science [8] with clear interpretations of uncertainty, which is why it is especially important for scientists to help this process by testing hypotheses with data. In making decisions, policymakers are inevitably faced with a cost–benefit balance. Illustrating the magnitude of focal effects is important for facilitating this process. As models are relied upon increasingly for public education and decision-making, presenting model results comprehensively and objectively is a responsibility that scientists should not take lightly.

## Supplementary Material

SI Text & Results

## Supplementary Material

SI Rcode
