# Supplementary material for: Comment on: ‘Blood does not buy goodwill: allowing culling increases poaching of a large carnivore’
Source: Proc Biol Sci. 2017 Mar 22;284(1851):20161459. doi: 10.1098/rspb.2016.1459 (PMC5378070; doi:10.1098/rspb.2016.1459)
Supplement: SI Text & Results [file rspb20161459supp1.pdf]

Supplemental Information: Comment on Chapron and Treves 2016

Kim M. Pepin, Shannon L. Kay, Amy J. Davis

1 Model Specification

Our interpretation of the model presented in Chapron and Treves (2016) and implemented by us here is

$$N_{t+1} \sim \text{LogNorm}(\mu_t^S, \sigma_{proc}) \quad (1)$$

$$\mu_t^S = \text{Log}(N_t^S e^{r_{t+1}^S} - \gamma H_{t+1}^S) \quad (2)$$

$$r_{t+1}^S = \beta_0^S + \beta_1 D_{t+1}^S \quad (3)$$

$$N_{obsMin,t}^S \sim \text{Pois}(0_{min} \psi_t^S) \quad (4)$$

$$N_{obsMax,t}^W \sim \text{Pois}(0_{max} \psi_t^S) \quad (5)$$

$$\psi_t^S \sim \text{Gamma}\left(\left(\frac{N_t^S}{\sigma_{Nobs}^S}\right)^2, \frac{N_t^S}{(\sigma_{Nobs}^S)^2}\right) \quad (6)$$

$$\beta_0^S \sim \text{N}(0, 100000) \quad (7)$$

$$\beta_1 \sim \text{N}(0, 100000) \quad (8)$$

$$\sigma_{Nobs}^S \sim \text{Unif}(0, 100) \quad (9)$$

$$\sigma_{proc} \sim \text{Unif}(0, 0.5) \quad (10)$$

$$O_{min} \sim \text{N}(1, 100000) \quad (11)$$

$$O_{max} \sim \text{N}(1, 100000) \quad (12)$$

where N_{t+1} are the predicted population counts with a deterministic mean based on the amount of growth ($e^{r_{t+1}^S}$) and culling (H_{t+1}^S) with the growth rate being linearly dependent on the proportion of days the policy was in effect (D_{t+1}). Observed counts $N_{obsMin,t}^S$ and $N_{obsMax,t}^W$ followed a Poisson distribution with means proportional to ψ_t^S, where ψ_t^S was Gamma distributed with a mean of N_t^S and standard deviation σ_{Nobs}^S. Non-informative priors were used for the linear growth parameters β_0^S and β_1 and state-specific standard

deviation parameters σ^S_{Nobs}. A more informative uniform(0,0.5) prior was used for the standard deviation of predicted population counts, and the scaling parameters O_{min} and O_{max} both had Gaussian priors with a mean of 1 and large variance. However, these scaling parameters were constrained as in Chapron and Treves (2016) with $O_{min} \in [0, 1]$ and $O_{max} \in [1, 10.]$

In order to re-create the model specified in Chapron and Treves (2016) we confirmed the following details with the authors and implemented them as described below:

- In the data set cited in Chapron and Treves (2016) the parameter D_t was given as the number of days in year t that the policy was active. In fact, these data were included in the model as the proportion of days within wolf year t.

- As the authors state in the main text σ_{Nobs} and ψ^S_t both varied by state.

- The order of biological events in the model is as follows: counting, population growth (births), then culling. The count information comes from late winter in the previous wolf year. Then births happen at the beginning of the wolf year in mid April. The model assumes that all culling happens after the population growth but before the end-of-wolf-year count.

- The prior distributions for O_{min} and O_{max} are irregular with identical priors that have a mean equal to 1. However, O_{min} is constrained to be within [0,1] and O_{max} is similarly constrained to be within [1,10], which are equivalent to uniform(0,1) and uniform(1,10) prior distributions. These are the prior distributions we used in our version of the model.

- Initial N_t (or equivalently μ_t) was randomly chosen using a prior distribution of $N_t \sim \text{gamma}(10^6, 10^6)$.

- The value μ^S_t was constrained so that $(N^S_t e^{r^S_{t+1}} - \gamma H^S_{t+1}) > 0$.

- There were three values in Wisconsin from 2007-2009 in the Rdata file provided by the authors that are not equal to the counts provided in their reference `https://www.fws.gov/midwest/wolf/aboutwolves/pdf/MN-WI-MI-table.pdf`. This is because the data used for the study were taken from Wydeven et. al. 2009 until 2007 and the Wisconsin DNR annual reports after that. We are not sure why these values differ, but we used the same values as Chapron and Treves (2016) provided in their R file.

- Chapron and Treves (2016) included some additional data sources (pack size, pack reproduction probability and area covered by packs) in their model to account for possible negative density-dependent effects due to culling actions. These effects were implemented directly on N_t, outside the main equation for population growth. We did not include these effects in our version of the model because the

posterior distributions reported by Chapron and Treves (2016) for each of these parameters was 0 ± 0, indicating that they were insignificant in the model structure.

1.1 Model implementation

To verify that our implementation matched that of (R Core Team, 2015), we implemented the model (as specified above) using two independent methods: 1) using JAGS software with the *rjags* package (Plummer, 2016) (as the authors did), and 2) by developing our own MCMC sampler in R (R Core Team, 2015). The purpose of the second method was because we were more comfortable with this approach as it gives the user more control and understanding over what the sampler is doing and we thought it might be appealing to others who would like more flexibility with implementation of similar models. Details of the custom sampler are provided in SI R code. We ran ten Monte Carlo Markov chains (100,000 iteration each, with a burn in of 20,000 iterations). We assessed convergence graphically for each parameter. We calculated the posterior median and standard deviation values to match Chapron and Treves (2016).

1.2 Simulations

We demonstrated differences in biological strength and statistical strength by using posterior distributions for β_1 (the policy effect parameter) as in Chapron and Treves (2016). β_1 is distributed normally, with a mean = -0.03 SD=0.03 (from Chapron and Treves (2016)). We used these values as our starting point. We examined the impacted of a more biologically meaningful effect (β_1 = -0.3) and used a standard deviation that matched Chapron and Treves (2016) in at 83% negative effect. We examined both the weak (β_1 =-0.03) and strong (β_1 =-0.3) biological effect with a weak (83% negative) and strong (99% negative) statistical effect. The distributions are shown in Figure 2 from the main text.

To show the impact to the population with these four different cases of biological and statistical effects, we estimated the population size with and without a policy effect by drawing N_t values from $N_t^S e^{r_{t+1}^S} - \gamma H_{t+1}^S$. Where $r_t = \beta_0 + \beta_1 * D$ for the policy effect model and $r_t = \beta_0$ for the model without a policy effect. The mean and standard errors were used from Chapron and Treves (2016) to draw from their given distributions to get values for β_0, β_1, and γ. The mean and credible intervals for the population estimates were calculated based on 1000 simulations for each condition.

2 Results

Below, we report median and 95% credible intervals for the two methods we used to implement the Chapron and Treves (2016) model. Note, while we captured the median values presented in Chapron and Treves

(2016) closely, our credible intervals tended to be larger (Table S1). We feel this makes sense based on the amount of data used. Also, the custom sampler captured the values presented by Chapron and Treves (2016) more closely than the JAGS version thus we used the custom sampler model for all analyses.

Table S1: Posterior parameter estimates from JAGS and custom MCMC models.

	JAGS		Custom MCMC	
	median ± SD	95% credible interval	median ± SD	95% credible interval
β_0^M	0.16 ± 0.41	(-0.65, 0.96)	0.15 ± 0.03	(0.08,0.22)
β_0^W	0.19 ± 0.41	(-0.61, 0.99)	0.18 ± 0.04	(0.10, 0.25)
β_1	-0.03 ± 0.69	(-1.37, 1.34)	-0.03 ± 0.08	(-0.19, 0.12)
γ	1.05 ± 0.27	(0.53, 1.58)	1.03 ± 1.08	(-1.53, 3.10)
0_{max}	1.03 ± 0.02	(1.00, 1.08)	1.26 ± 0.02	(1.23, 1.29)
0_{min}	0.97 ± 0.02	(0.93, 1.00)	0.99 ± 0.01	(0.98,1.00)
σ_{Nobs}^M	30.72 ± 22.80	(1.63,86.51)	11.71 ± 8.93	(0.92, 34.37)
σ_{Nobs}^W	32.81 ± 23.83	(1.83, 89.45)	10.42 ± 9.13	(0.82, 34.68)
σ_{proc}	0.48 ± 0.03	(0.39, 0.50)	0.11 ± 0.04	(0.07, 0.21)

Posterior distribution for the policy effect parameter by the JAGS and custom-made version of the model. Note we were not able to reproduce results with such narrow credible intervals as in the fits from Chapron and Treves (2016) (Figs. S1 and S2). While the β_1 posterior distribution in Chapron and Treves (2016) overlapped zero by only 17%, in our results the posterior distribution overlapped zero by 48% for the JAGS results and 38% for the custom sampler.

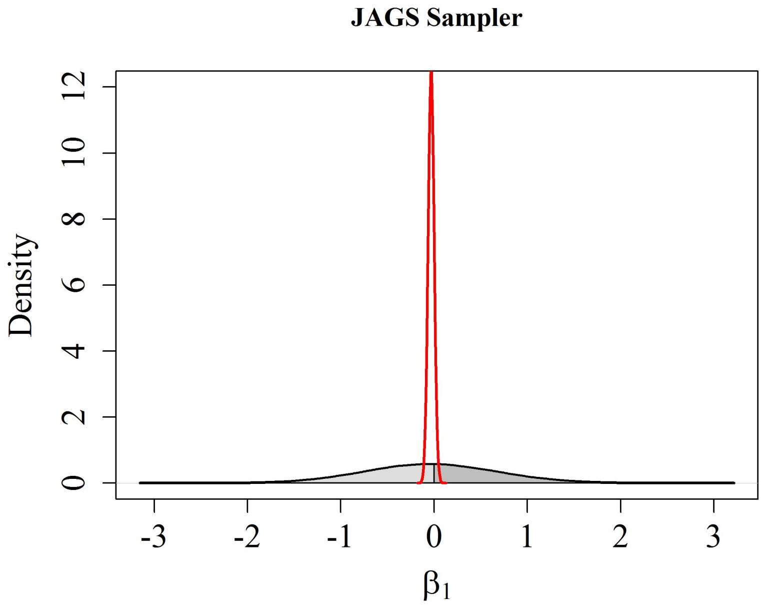

Figure S1: Posterior distribution of β_1 from the JAGS sampler with light grey shading below zero (representing negative policy effect) and darker grey shading above zero (positive policy effect). The red line represents the posterior distribution of Chapron and Treves (2016).

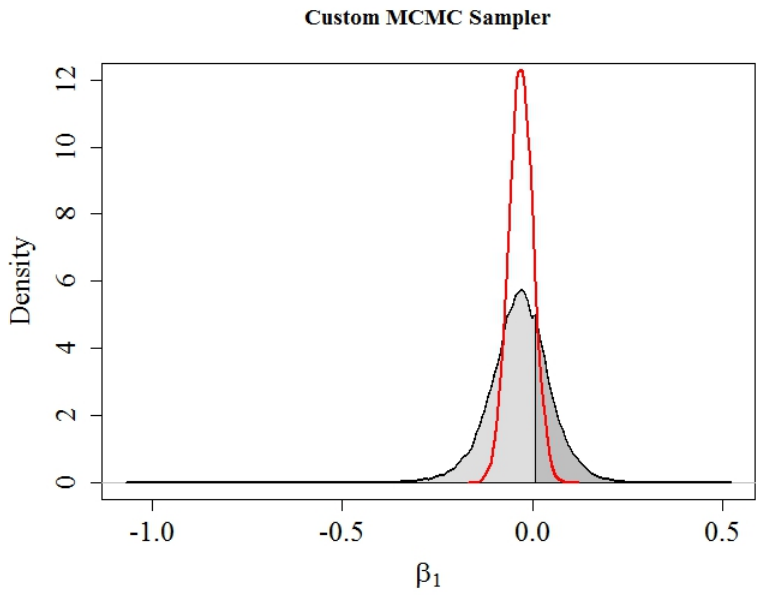

Figure S2: Posterior distribution of β_1 from our custom MCMC sampler written in R with light grey shading below zero (representing negative policy effect) and darker grey shading above zero (positive policy effect). The red line represents the posterior distribution of Chapron and Treves (2016).

On a side note, when we estimate Nt from the fitted model with and without a policy effect and calculate the mean number of wolves different in the two models (e.g., impact of the policy effect in terms of the number of wolves), there were 2.33 wolves fewer in Michigan but 0.11 wolves more in Wisconsin. These results are further evidence that the finding of a negative median value for the policy effect is not robust.

3 Order of biological events

Upon initial analyses with the data provided by Chapron and Treves (2016), we were concerned the biology could be misrepresented in the model specification. Correct model specification depends on the order of culling, counting and growth events throughout the wolf year (April 15th-April 14th; Fig. S3). As counts occurred in winter (defined as 09/15 to 04/14 in Wydeven et al. (2009) and growth in early spring (03/15-05/31), thus counting is the last event in the specified wolf year. Thus, if all data were organized by wolf year the order of events would be growth, culling, and counting, suggesting that the correct model specification would be $N_{t+1} = N_t e^{r_{t+1}} \gamma H_{t+1}$ (in contrast to $N_{t+1} = N_t e^{r_t} \gamma H_t$ as specified in Chapron and Treves (2016)) in order to not double count culls in the prediction of N_{t+1} (Fig. S3). We checked the data organization provided by the authors in an R file and noticed that the data were indeed organized in this way (i.e., counting and reproduction as the last event in the wolf year). However, when we ran

the correct model ($N_{t+1} = N_t e^{r_{t+1}} \gamma H_{t+1}$), we recovered a posterior distribution for the policy effect that was even more like the authors (median = -0.03 instead of -0.07) suggesting they may have run the correct model but simply misspecified it. Nonetheless, thinking about the order of biological events in the model specification, highlighted a potentially important point for us. Based on the time frames of legal culling, which occurred during any time of the year, it is likely that in some years most of the culling occurred at different times relative to the other events, such that the model was incorrectly specified in some years but not others. As predicted abundance changes by the different model structures (Fig. S4), temporal variation in misspecification could explain some of the variation in the policy effect. To improve application of this type of approach, data on the order of events for each year should be carefully considered in the model specification.

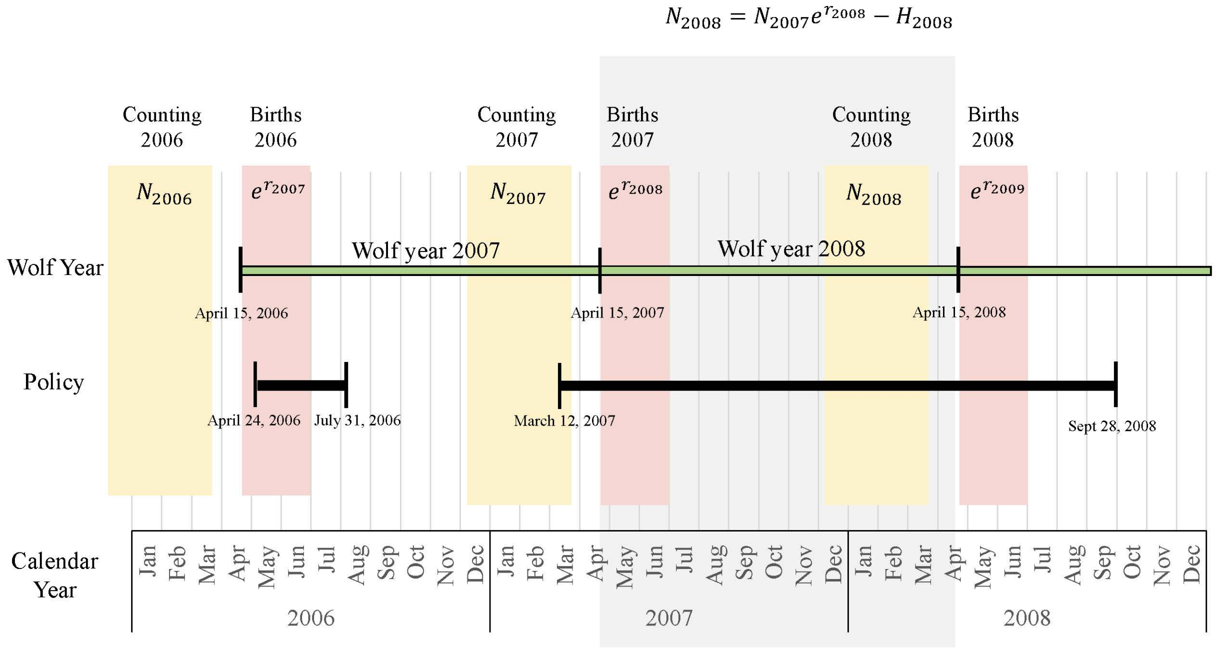

Figure S3: Schematic demonstrating the order of biological events relative to the calendar year and wolf year (April 15-April 14). Counts of wolf populations were conducted in the winter (Wydeven et al. (2009)), and births occured in the spring (Wydeven et al. (2009)). To demonstrate focus on wolf year 2008 (shaded in grey). The order of biological event in the model Chapron and Treves (2016) describe are: counting, births, then culling. To estimate the abundance for wolf year 2008, we need to use the count data from 2007, the birth data from 2008, and the culling data from 2008. Thus the correct model specification is $N_{t+1} = N_t e^{r_{t+1}} \gamma H_{t+1}$.

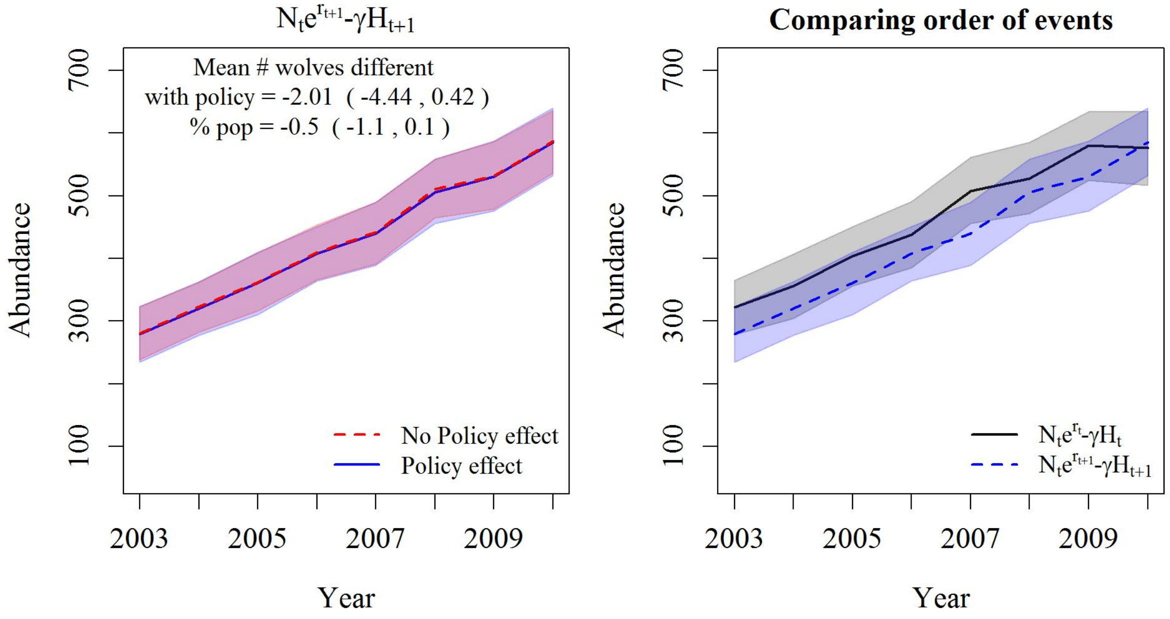

Figure S4: Abundance estimates with 95% CIs for different order of events, showing results for Michigan. A) Shows the abundance with and without a policy effect for $N_{t+1} = N_t e^{r_{t+1}} \gamma H_{t+1}$. B) Shows the abundance estimates with a policy effect for $N_{t+1} = N_t e^{r_t} \gamma H_t$ (blue) and estimate from $N_{t+1} = N_t e^{r_{t+1}} \gamma H_{t+1}$ (green).

4 Other models

An important biological feature of wolf population dynamics which was overlooked in the model specification Chapron and Treves (2016) is that wolf populations do not grow without bounds. There is substantial evidence that population abundance is limited by territoriality, social behavior and environmental conditions [Smith et al. (2015), Stahler et al. (2013),Cassidy et al. (2015), Cubaynes et al. (2014)]. To investigate implications of this assumption we additionally fit models that assumed logistic growth and compared them to those assuming exponential growth (Table S2). Although the median values for the policy effect tended to be less negative in the logistic growth models relative to the exponential growth models, the two versions were not statistically different. This could be because the wolf population has apparently been experiencing a linear increase in population growth over the time frame of these analyses, making the exponential model a reasonable approximation for the population trajectory. However, the lack of difference between the models does not preclude the potential importance of density-dependent growth under some biological conditions.

In the model of Chapron and Treves (2016), the potential for negative density-dependent feedbacks from culling were explored using covariate data (pack size, pack reproduction probability and area covered by packs) which entered the model at a hierarchical level that was much less significant than how the policy effect was considered. Thus, we looked at one of these factors (pack size) by entering it as a linear effect on

growth rate, the same way the policy effect is entered to allow for a more direct comparison of the potential influence of this variable. A table of model results comparing the models we examined is shown in Table S2.

Pack size was not a strong effect ($\beta_1 = -0.005 \pm 0.04$) on growth rate and the DIC value for that model was higher than others examined. This may, in part, be due to the lack of variability in the mean pack size parameter over time (Fig. S5). Additionally, this information was only available for Wisconsin and thus the power to detect an effect may be lower than the policy effect, if a true effect did exist.

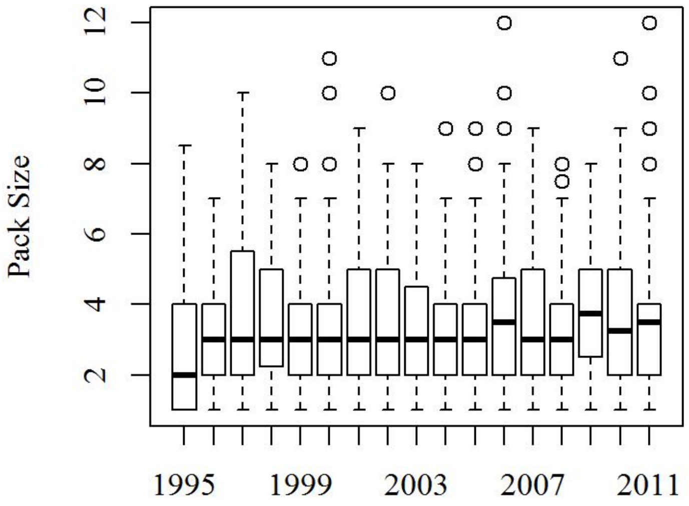

Figure S5: Boxplot of the pack sizes by year observed in Wisconsin.

Another hypothesis that Chapron and Treves (2016) examined was an impact of area covered by packs on population size. The potential role of this factor in negative density-dependence is not entirely clear, as increased area could be a consequence of increased abundance. Thus we did not test this in an alternative model structure. In Fig. S6 we show that there is indeed a strong correlation between the area factor and abundance. However, Chapron and Treves (2016) estimated an effect on abundance as 0 ± 0 which is not consistent with this relationship.

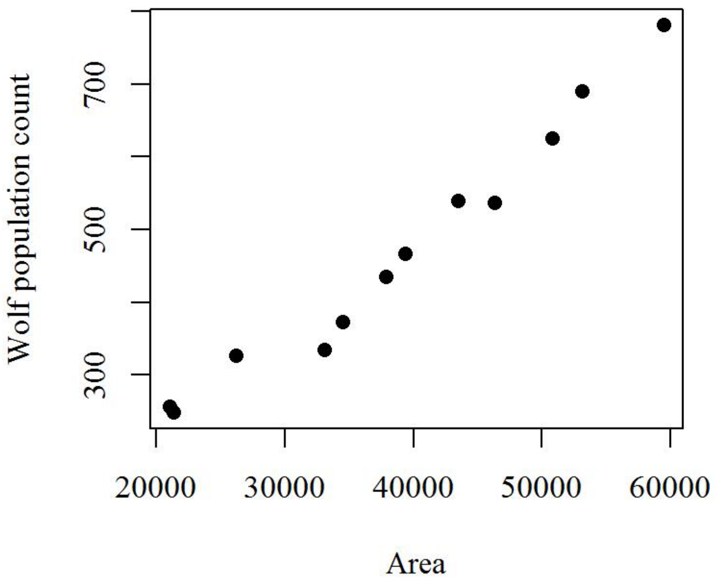

Figure S6: Plot of minimum wolf population count by area in Wisconsin.

Table S2: Table of model results for different growth types and models on growth.

Model description	Growth Type	Model on Growth	DIC	Posterior β_1 Median $\pm$ SD	95% CI
Exponential growth with a policy effect	$N_{t-1}^S e^{r_t^S} - \gamma H_t^S$	$r_t^S = \beta_0^S + \beta_1 D_t^S$	475.74	$\beta_1 = -0.03 \pm 0.08$	(-0.19, 0.12)
Exponential growth without a policy effect	$N_{t-1}^S e^{r_t^S} - \gamma H_t^S$	$r_t^S = \beta_0^S$	474.96		
Logistic growth with a policy effect	$\frac{N_{t-1}^S e^{r_t^S}}{1+\left(\frac{N_{t-1}^S}{K^S}\right)(e^{r_t^S}-1)} - \gamma H_t^S$	$r_t^S = \beta_0^S + \beta_1 D_t^S$	476.66	$\beta_1 = 0.003 \pm 0.14$	(-0.28, 0.27)
Logistic growth without a policy effect	$\frac{N_{t-1}^S e^{r_t^S}}{1+\left(\frac{N_{t-1}^S}{K^S}\right)(e^{r_t^S}-1)} - \gamma H_t^S$	$r_t^S = \beta_0^S$	475.79		
Exponential growth with a pack size effect	$N_{t-1}^S e^{r_t^S} - \gamma H_t^S$	$r_t^S = \beta_0^S + \beta_1 P_t^S$	476.74	$\beta_1 = 0.01 \pm 0.04$	(-0.05, 0.08)
Exponential growth with policy and pack size effect	$N_{t-1}^S e^{r_t^S} - \gamma H_t^S$	$r_t^S = \beta_0^S + \beta_1 D_t^S + \beta_2 P_t^S$	475.86	$\beta_1 = -0.016 \pm 0.09$<br>$\beta_2 = -0.012 \pm 0.03$	(-0.20, 0.14)<br>(-0.06, 0.08)

References

Kira A Cassidy, Daniel R MacNulty, Daniel R Stahler, Douglas W Smith, and L David Mech. Group composition effects on aggressive interpack interactions of gray wolves in yellowstone national park. *Behavioral Ecology*, 26(5):1352–1360, 2015.

Guillaume Chapron and Adrian Treves. Blood does not buy goodwill: allowing culling increases poaching of a large carnivore. In *Proc. R. Soc. B*, volume 283, page 20152939. The Royal Society, 2016.

Sarah Cubaynes, Daniel R MacNulty, Daniel R Stahler, Kira A Quimby, Douglas W Smith, and Tim Coulson. Density-dependent intraspecific aggression regulates survival in northern yellowstone wolves (canis lupus). *Journal of Animal Ecology*, 83(6):1344–1356, 2014.

Martyn Plummer. *rjags: Bayesian Graphical Models using MCMC*, 2016. URL `http://CRAN.R-project.org/package=rjags`. R package version 4-6.

R Core Team. *R: A Language and Environment for Statistical Computing*. R Foundation for Statistical Computing, Vienna, Austria, 2015. URL `https://www.R-project.org/`.

Douglas W Smith, Matthew C Metz, Kira A Cassidy, Erin E Stahler, Richard T McIntyre, Emily S Almberg, and Daniel R Stahler. Infanticide in wolves: seasonality of mortalities and attacks at dens support evolution of territoriality. *Journal of Mammalogy*, 96(6):1174–1183, 2015.

Daniel R Stahler, Daniel R MacNulty, Robert K Wayne, Bridgett VonHoldt, and Douglas W Smith. The adaptive value of morphological, behavioural and life-history traits in reproductive female wolves. *Journal of Animal Ecology*, 82(1):222–234, 2013.

Adrian P. Wydeven, Jane E. Wiedenhoeft, Ronald N. Schultz, Richard P. Thiel, Randy L. Jurewicz, Bruce E. Kohn, and Timothy R. Van Deelen. *History, Population Growth, and Management of Wolves in Wisconsin*, pages 87–105. Springer New York, New York, NY, 2009. ISBN 978-0-387-85952-1. doi: 10.1007/978-0-387-85952-1_6. URL `http://dx.doi.org/10.1007/978-0-387-85952-1_6`.
