# Supplementary material for: Comment on: ‘Blood does not buy goodwill: allowing culling increases poaching of a large carnivore’
Source: Proc Biol Sci. 2017 Mar 22;284(1851):20161459. doi: 10.1098/rspb.2016.1459 (PMC5378070; doi:10.1098/rspb.2016.1459)
Supplement: SI Rcode [file rspb20161459supp2.pdf]

Supplemental Information: R Code for comment on Chapron and Treves 2016

Amy Davis and Shannon Kay

JAGS Sampler

```
library(rjags)

library(coda)

load("chapron_treves_2016.Rdata")


Hm=michigan$H

Hw=wisconsin$H

ym=michigan$Nobs

yw=wisconsin$Nobs.Min

ywmax=wisconsin$Nobs.Max

Dm=treatment$days_cull_MI/365

Dw=treatment$days_cull_WI/365


modelstuff<-"

model{

for(i in 1:t){

Nm[i+1]~ dlnorm(mum[i],sigproc)

mum[i]=log(mum.term[i]*mum.term.ind[i]+0.0001)

mum.term.ind[i]<-ifelse(mum.term[i]>1,1,0)

mum.term[i]<-Nm[i]*exp(rm[i+1])-gam*Hm[i+1]

rm[i+1]<-b0m+b1*Dm[i+1]

ym[i]~dpois(omin*psim[i])
```

```
psim[i]~dgamma(alpham[i],betam[i])
alpham[i]<-pow(Nm[i]/sigobsm,2)
betam[i]<-Nm[i]*pow(sigobsm,-2)
#
Nw[i+1]~ dlnorm(muw[i],sigproc)
muw[i]=log(muw.term[i]*muw.term.ind[i]+0.0001)
muw.term.ind[i]<-ifelse(muw.term[i]>1,1,0)
muw.term[i]<-Nw[i]*exp(rw[i+1])-gam*Hw[i+1]
rw[i+1]<-b0w+b1*Dw[i+1]
yw[i]~dpois(omin*psiw[i])
ywmax[i]~dpois(omax*psiw[i])
psiw[i]~dgamma(alphaw[i],betaw[i])
alphaw[i]<-pow(Nw[i]/sigobsw,2)
betaw[i]<-Nw[i]*pow(sigobsw,-2)
}
gam~dnorm(1.06, 14)
tau<-10E-6
b0m~dnorm(0,tau)
b0w~dnorm(0, tau)
b1~dnorm(0, tau)
omin~dnorm(1,tau)I(0,1)
omax~dnorm(1,tau)I(1,10)
sigobsw~dunif(0,100)
sigobsm~dunif(0,100)
sigproc~dunif(0,0.5)
Nw[1]~ dgamma(1E-6,1E-6)
Nm[1]~ dgamma(1E-6,1E-6)
}
"

model.spec<-textConnection(modelstuff)
model <- jags.model(model.spec,
data = list('ym'=ym,'Dm'=Dm,'Hm'=Hm,'yw'=yw,'Dw'=Dw,'Hw'=Hw,'ywmax'=ywmax,'t'=17),
```

```
n.chains=8,
n.adapt=100)
update(model,n.iter=50000)
output=coda.samples(model=model,
        variable.names=c("Nw","Nm","gam","b0m","b0w","b1","omin","omax","sigobsw","sigobsm","sigproc"),
        n.iter=100000,thin=10)
```

Custom Sampler

```
wolves=data.frame(State=c(rep("MI",17),rep("WI",17)),Year=c(michigan$year[2:18],wisconsin$year[2:18]),
        Nobs=c(michigan$Nobs[1:17],wisconsin$Nobs.Min[1:17]),H=c(michigan$H[2:18],wisconsin$H[2:18]),
        D=c(treatment$days_delist[2:18]/365))
wi.max=wisconsin$Nobs.Max

wolf.mcmc<-function(wolves,ymax,mutune,psi.tune,b0.tune,b1.tune,gam.tune,omin.tune,sigp.tune,sign.tune,n
##  wolves = dataframe from C&T with wolf data including year, min, removed, policy
##  ymax = maximum wolf count from Wisconsin only
##  mutune = tuning parameter for mu0
##  psi.tune = tuning parameter for psi
##  b0.tune = tuning for betas
##  b1.tune = tuning for b1
##  gam.tune = tuning for gamma
##  omin.tune = tuning for omin
##  sigp.tune = tuning for sig proc
##  sign.tune = tuning for sig.n
##  n.mcmc = the number of Gibbs iterations to run




###
### Libraries and functions
###
t_col <- function(color, percent = 30, name = NULL) {
```

```
#           color = color name
#         percent = % transparency
#            name = an optional name for the color

## Get RGB values for named color
rgb.val <- col2rgb(color)

## Make new color using input color as base and alpha set by transparency
t.col <- rgb(rgb.val[1], rgb.val[2], rgb.val[3],
max = 255,
alpha = (100-percent)*255/100,
names = name)

## Save the color
invisible(t.col)

}

###
### Data
###
T=length(wolves$State)/2


###
### Starting values
###
y=matrix(wolves$Nobs,T,2)
n.t=matrix(wolves$Nobs,T,2)
n.tstar=n.t
psi=n.t
psi.star=psi
```

```
mu0=y[1,]
#gam=rnorm(1,1.06,sqrt(1/14))
b0=c(0.14,0.16)
b1=rnorm(1,0,1)
sig.proc=0.06
sig.n=c(3.82,4.72)
omin=0.97
omax=1.03
### Derived
D=matrix(wolves$D,T,2)
H=matrix(wolves$H,T,2)
r=t(b0+t(b1*D))
mu=log(n.t*exp(r)-gam*H)




###
### Set up matrices to save iterations from the Gibbs sample
###

nsave=array(0,c(T,2,n.mcmc))  ### Abundance for each site
muosave=matrix(0,2,n.mcmc)
psisave=array(0,c(T,2,n.mcmc))
rsave=array(0,c(T,2,n.mcmc))

### Shared
ominsave=rep(0,n.mcmc)
omaxsave=rep(0,n.mcmc)
gamsave=rep(0,n.mcmc)
sigprocsave=rep(0,n.mcmc)
signsave=matrix(0,2,n.mcmc)
b0save=matrix(0,2,n.mcmc)
b1save=rep(0,n.mcmc)
```

```
n.burn=round(n.mcmc/10)
devsave=rep(0,n.mcmc)

###
### MCMC loop
###

for(l in 1:n.mcmc){
if(l%%100==0){cat(l," ")}

################
### Do each state separately

#####
##### Metropolis Hastings step for Nt
#####
### Generating proposal values using a log normal with the mean as the previous theta and variance is th


#### Do t=1 first
n.tstar=matrix(rpois((T)*2,n.t),T,2)

alpha=(n.t/sig.n)^2
beta=n.t/(sig.n^2)

alphastar=(n.tstar/sig.n)^2
betastar=n.tstar/(sig.n^2)

mu=log(rbind(mu0,n.t[-T,])*exp(r)-gam*H)
if(sum(ifelse(rbind(mu0,n.tstar[-T,])*exp(r)>gam*H,0,1))==0){

mu.star=log(rbind(mu0,n.tstar[-T,])*exp(r)-gam*H)
```

```
mh1=(dgamma(psi,alphastar,betastar,log=TRUE))+dlnorm(n.tstar,mu.star,sig.proc,log=TRUE)+dpois(n.t,n.tsta
mh2=(dgamma(psi,alpha,beta,log=TRUE))+dlnorm(n.t,mu,sig.proc,log=TRUE)+dpois(n.tstar,n.t,log=TRUE)


mhratio=exp(mh1-mh2)
tmp.keep=mhratio>runif(T*2)
n.t[tmp.keep]=n.tstar[tmp.keep]
n.t
}
### Updating
alpha=(n.t/sig.n)^2
beta=n.t/(sig.n^2)
mu=log(rbind(mu0,n.t[-T,])*exp(r)-gam*H)


############################
####
#### Sample from sig.proc
####
############################


sigproc.star=rbeta(1,(sigp.tune)/(1/sig.proc-1),sigp.tune)

mh1s=sum(dlnorm(n.t,mu,sigproc.star,log=TRUE))+dunif(sigproc.star,0,0.5,log=TRUE)+dbeta(sig.proc,(6)/(1/
mh2s=sum(dlnorm(n.t,mu,sig.proc,log=TRUE))+dunif(sig.proc,0,0.5,log=TRUE)+dbeta(sig.proc,(6)/(1/sig.proc


mhratios=exp(mh1s-mh2s)
tmp.keep=mhratios>runif(1)
sig.proc[tmp.keep]=sigproc.star[tmp.keep]
sig.proc
```

```
###########################
####
#### Sample from psi
####
###########################

psi.star=matrix(rnorm(T*2,psi,psi.tune),T,2)

mh1p=dpois(y,omin*psi.star,log=TRUE)+cbind(0,dpois(ymax,omax*psi.star[,2],log=TRUE))+dgamma(psi.star,al
mh2p=dpois(y,omin*psi,log=TRUE)+cbind(0,dpois(ymax,omax*psi[,2],log=TRUE))+dgamma(psi,alpha,beta,log=TR

mhratiop=exp(mh1p-mh2p)
tmp.keep=mhratiop>runif(T*2)
psi[tmp.keep]=psi.star[tmp.keep]
psi


###########################
####
#### Sample from sig.n
####
###########################

sign.star=rnorm(2,sig.n,sign.tune)

if(max(sign.star)<=100&min(sign.star)>0){
alphastar=(n.t/sign.star)^2
betastar=n.t/(sign.star^2)

mh1sn=sum(dgamma(psi,alphastar,betastar,log=TRUE))+dunif(sign.star,0,100,log=TRUE)
```

```
mh2sn=sum(dgamma(psi,alpha,beta,log=TRUE))+dunif(sig.n,0,100,log=TRUE)

mhratiosn=exp(mh1sn-mh2sn)
tmp.keep=mhratiosn>runif(2)
sig.n[tmp.keep]=sign.star[tmp.keep]
}
sig.n

### Updating
alpha=(n.t/sig.n)^2
beta=n.t/(sig.n^2)
mu=log(rbind(mu0,n.t[-T,])*exp(r)-gam*H)


############################
####
#### Sample from omin
####
############################

omin.star=rbeta(1,1,1/omin-1)
omin.star=rnorm(1,omin,omin.tune)

if(omin.star<=1&omin.star>0){
mh1o=sum(dpois(y,omin.star*psi,log=TRUE))+dnorm(omin.star,1,sigb)
mh2o=sum(dpois(y,omin*psi,log=TRUE))+dnorm(omin,1,sigb)

mhratioo=exp(mh1o-mh2o)
tmp.keep=mhratioo>runif(1)
omin[tmp.keep]=omin.star[tmp.keep]
omin
}
```

```
###########################
####
#### Sample from omax
####
###########################

omax.star=rnorm(1,omax,omax.tune)


if(omax.star<=10&omax.star>1){
mh1o=sum(dpois(ymax,omax.star*psi,log=TRUE))+dnorm(omax.star,1,sigb)
mh2o=sum(dpois(ymax,omax*psi,log=TRUE))+dnorm(omax,1,sigb)

mhratioo=exp(mh1o-mh2o)
tmp.keep=mhratioo>runif(1)
omax[tmp.keep]=omax.star[tmp.keep]

}
omax

###########################
####
#### Sample from gamma
####
###########################

gam.star=rnorm(1,gam,gam.tune)


if(sum(ifelse(rbind(mu0,n.t[-T,])*exp(r)>gam.star*H,0,1))==0){
mu.star=log(rbind(mu0,n.t[-T,])*exp(r)-gam.star*H)

mh1g=sum(dlnorm(n.t,mu.star,sig.proc,log=TRUE))+dnorm(gam.star,1.06,sqrt(1/14))
mh2g=sum(dlnorm(n.t,mu,sig.proc,log=TRUE))+dnorm(gam,1.06,sqrt(1/14))
```

```
mhratiog=exp(mh1g-mh2g)
tmp.keep=mhratiog>runif(1)
gam[tmp.keep]=gam.star[tmp.keep]
gam
mu=log(rbind(mu0,n.t[-T,])*exp(r)-gam*H)
}



############################
####
#### Sample from bs
####
############################

b0.star=rnorm(2,b0,b0.tune)

rstar=t(b0.star+t(b1*D))
if(sum(ifelse(rbind(mu0,n.t[-T,])*exp(rstar)>gam*H,0,1))==0){

mu.star=log(rbind(mu0,n.t[-T,])*exp(rstar)-gam*H)

mh1b=sum(dlnorm(n.t,mu.star,sig.proc,log=TRUE))+dnorm(b0.star,0,sigb)
mh2b=sum(dlnorm(n.t,mu,sig.proc,log=TRUE))+dnorm(b0,0,sigb)

mhratiob=exp(mh1b-mh2b)
tmp.keep=mhratiob>runif(2)
b0[tmp.keep]=b0.star[tmp.keep]

}
b0
r=t(b0+t(b1*D))
mu=log(rbind(mu0,n.t[-T,])*exp(r)-gam*H)
```

```
#### B1
b1star=rnorm(1,b1,b1.tune)

rstar=t(b0+t(b1star*D))
if(sum(ifelse(rbind(mu0,n.t[-T,])*exp(rstar)>gam*H,0,1))==0){

mu.star=log(rbind(mu0,n.t[-T,])*exp(rstar)-gam*H)

mh1b=sum(dlnorm(n.t,mu.star,sig.proc,log=TRUE))+dnorm(b1star,0,sigb)
mh2b=sum(dlnorm(n.t,mu,sig.proc,log=TRUE))+dnorm(b1,0,sigb)

mhratiob=exp(mh1b-mh2b)
tmp.keep=mhratiob>runif(1)
b1[tmp.keep]=b1star[tmp.keep]
b1
}

r=t(b0+t(b1*D))
mu=log(rbind(mu0,n.t[-T,])*exp(r)-gam*H)

### Calculate deviance
devsave[l]=-2*(sum(dpois(y,omin*psi,log=TRUE))+sum(dpois(ymax,omax*psi[,2],log=TRUE)))


###############################
####
#### Save samples
####
###############################
#matplot(n.t,type="l",lwd=2)
```

```
nsave[,,l]=n.t
muosave[,l]=mu0
psisave[,,l]=psi
ominsave[l]=omin
omaxsave[l]=omax
b0save[,l]=b0
b1save[l]=b1
gamsave[l]=gam
sigprocsave[l]=sig.proc
signsave[,l]=sig.n
rsave[,,l]=r
}


list(b0save=b0save,b1save=b1save,gamsave=gamsave,nsave=nsave,omaxsave=omaxsave,ominsave=ominsave,
psisave=psisave,rsave=rsave,sigprocsave,sigprocsave,DIC=DIC)
}
```
